# Changes in Serum Leptin During Phases of Menstrual Cycle of Fertile Women: Relationship to Age Groups and Fertility

**DOI:** 10.5812/ijem.6872

**Published:** 2012-12-21

**Authors:** Olawole Micheal Ajala, Paul Sunday Ogunro, Gabriel Folorunsho Elusanmi, Olugbemiga Ebenezer Ogunyemi, Abidemi Abibat Bolarinde

**Affiliations:** 1 Department of Chemical Pathology, Lagos State Laboratory Services Lagos Island General Hospital, Lagos, Nigeria; 2 Department of Chemical Pathology, College of Health Sciences, Ladoke Akintola University of Technology, Osogbo, Nigeria; 3 Department of Chemical Pathology, College of Medicne, Ogun State University, Shagamu Ogun State, Nigeria; 4 Department of Obstetrics and Gynaecology, Federal Medical Center, Owo, Ondo State, Ngeria

**Keywords:** Leptin, FSH, LH, Progesterone, Oestradiol, Menstrual Cyc

## Abstract

**Background:**

It is established that serum level of leptin is affected by transitional phases of reproduction. It is also reported that the puberty is triggered when body fat and circulating levels of leptin exceed the critical thresholds, butthere is less focus on the serum level of leptin and its relationship with different phases of menstrual cycle and the fertility.

**Objectives:**

The present study try to determines the serum concentration of leptin and fertility hormonesin the various phases of normal menstrual cycle of fertile women and compare any difference in serum concentration between age groups of 18-30 years and 31-41 years It is a well known fact that fertility start to decrease from age 31 years.

**Patients and Methods:**

A total of 118 healthy fertile women with normal menstrual cycle aged between 18-40, were divided into two age groups (n = 65) 18-30 years and (n = 53) 31-40 years. Serum concentrations of leptin, estradiol, progesterone, luteinising hormone (LH) and follicle-stimulating hormone (FSH) were measured on day1 (menstrual phase), day7 (proliferative/follicular phase), day14 (ovulatory phase), day21 (luteal phase) and day 28(secretory phase) of the menstrual cycle.

**Results:**

There was a significant increase (P < 0.05) in leptin levels on day 14 (12.75 + 5.8 ng/mL) and day 21 (12.91 + 3.2 ng/mL) for age group 18-30 years compared to day 14 (11.60 + 3.2 ng/mL) and day 21 (11.60 + 3.2 ng/mL) for age group 31-40 years. Leptin was positively correlated with FSH on day 14, with LH on day 7 and day 21; likewise, with progesterone on day 21 and day 28 and with estradiol on day 7 and day 14 for both age groups.

**Conclusions:**

The serum leptin level was at the lowest level during the menstrual and secretory phase and the highest level was around the luteal phase. The significant increase ofleptin in the younger age group raise this question whether circulating leptin has any role to play in the age of pregnancy and fertility. Data in this study shows that leptin level was affected with increase in age; therefore changes in leptin level will affect fertility in this study suggest that there may be a relation between leptin levels and fertility.

## 1. Background

Leptin is the hormone product of the LEP gene and was originally thought to be produced only by the adipocytes to modulate satiety and energy homeostasis (1, 2). However, thispolypeptide is known as a product of many tissues like gastric mucosa, mammary epithelial cell, human placenta and uterine endometrium now and enhanced levels are associated with the advent of reproductive maturity and fertility (3, 4). In humans, serum leptin concentration correlates with the adiposity measured either as the body mass index (BMI) or as percentage of the body fat (5, 6). A Study also revealed that the mean serum level of leptin is two to three times higher in women than men with the same body fat mass (7). In females, menarche was shown to be more closely related to fat distribution than skeletal maturity and it is more related with the lower-body (gluteofemoral) fat than the upper-body fat; therefore, blood leptin levels are much more strongly related to gluteofemoral than upper-body fa suggesting that leptin may convey information about the fat distribution to the hypothalamus during the puberty (8). The differences in blood leptin levels between upper and lower-body fat distribution can be attributed to ob gene of adipocytes in various fats.

Infertility is a worldwide problem, according to World Health Organization (WHO) and Demographic Health Surveys (DHS) comparative report (9) one in four ever-married women of the reproductive age in the developing countries are infertile because of primary or secondary infertility. The prevalence of primary infertility in sub-Saharan Africa is relatively low and it exceeds 3% in less than a third of the 28 African countries analysed (10). However, the prevalence of primary infertility in southwestern part of Nigeria is 22.5% (11). Leptin receptors have been identified in hypothalamus, gonadotrope cells of the anterior pituitary (12), granulose, theca and interstitial cells of ovary (13) as well as in the endometrium (14), all of which are concerned with fertility.

In general, the menstrual cycle, which lasted for 21–35 days, results in the development and release of a mature egg for fertilization. During the follicular phase of the menstrual cycle, follicle-stimulating hormone (FSH) stimulates the differentiation and proliferation of granulosa cells in ovarian follicles from approximately 1 million to 50 million cells (15). This marked increase in cell number is due to the FSH-stimulated transcription of genes that encode growth factors, such as insulin-like growth factor 1 (IGF-1), that function in both a paracrine and autocrine fashion (16). As the granulosa cells secrete both estrogen and progesterone, the less mature FSH-dependent follicles become atretic. The dominant follicle, which is selected by the mid-follicular phase, secretes increasing amounts of estrogen, which exerts a negative feedback on FSH and LH secretion (15).

Study showed that oestradiol may stimulate the production of leptin from the adipocytes and that leptin could act in the pituitary ovarian axis during fasting to improve reproductive function by partly stimulating estrogen secretion (17). A possible direct intra-ovarian effect of leptin was also noted in a study on rats in which the action of insulin-like growth factor (IGF-1) and FSH on oestradiol production by granulosa cells cultured was attenuated by the addition of leptin (18). In a recent study, serum leptin was found to be significantly correlated with serum progesterone and estrogen (19). There is hypothesis that the central target for leptin is Gonadotropin Releasing Hormone (GnRH) neurons in the hypothalamus, investigating this hypothesis both intact hemihypothalamic explants and enzymatically dispersed hypothalamic neurons in a perifusion system demonstrate that leptin plays a direct stimulatory role in the regulation of GnRH release (20). Study also show that the major change following leptin administration is normalization of menses without a change in ovarian size, which is most consistent with the effect of leptin to regularize normal pulsatile LH secretion; allowing for the full agonistic effect of the gonadotropin on the ovary (21). Results published on serum leptin levels during the physiological menstrual cycle vary considerably. Some studies showed significant increase in the late follicular phase (22, 23), and some did so on the day of the onset of the luteinising hormone (LH) surge (24, 25). Other investigations described significant increase in serum leptin levels in the late luteal phase (26, 27), and again others reported only small, not statistically significant variations during the menstrual cycle (28, 29).

## 2. Objectives

Since fewer studies had been carried out on the existance of any relationship between levels of the fertility of a normal menstruating women based on the age and the leptin level along with estradiol, progesterone, LH and FSH, this study aimed at examining the changes in the serum leptin levels during the various phases of the menstrual cycle and investigating the differences in the serum leptin concentrations between two age groupsof 18-31 and 31-41 ; and if there is any relationship between leptin and fertility hormones (30).

## 3. Patients and Methods

### 3.1. Subjects

The proposed study was carried out at the department of Obstetrics and Gynaecology of the Lagos Island Hospital in Lagos Metropolis of Lagos State, Southwest of Nigeria, between September 2010 and August 2011. Lagos Metropolis is the commercial nerve center of Nigeria, which comprises various ethnic communities and majorities are Christians and Muslims. 

After the approval of the ethic committee of the institution, 160 fertile women who consented to participate in the study, the research procedure were explained to them. They were selected by convenient sampling methods. Inclusion criteria were healthy, sedentary, non-obese (BMI < 24), waist to hip circumference ratio of < 0.8, regular menstrual cycles (28 ± 2 days during the last 6months), never been previously treated with estrogens and/or progestins and had not taken any medication that might affect their hormonal level in the preceding 4weeks. All subjects were screened by medical history, physical examination; waist/hip circumference ratio and BMI were calculated. Exclusion criteria include those who are uncertain of their menstrual status, history of diabetic, hypertension, BMI > 24, thyroid and any endocrine disorder. One hundred and eighteen (118) women that fulfilled the inclusion criteria out of 160 were finally selected for the research and they were between the ages of 18-40 years. The selected women divided into two age groups of 18-30years (n = 65) and 31-40 years (n = 53). Blood samples were collected at 08:30 , after a 12 hr overnight fasting, on day 1 from the beginning of menstruation, and then every 7th day for 4 weeks (on days 7, 14, 21and 28) on plain bottle. Blood samples were allowed to clot, then the separated the serums were stored below -20oC until they were analyzed for leptin, oestradiol, progesterone, FSH and LH.

### 3.2. Analytical Procedures

Serum leptin was measured using the Assay Design Inc. Human Leptin Enzyme (cat # 1742-6 Human leptin Enzyme-linked immunosorbent ELISA Kit) (31) with inter- and intra-assay coefficients of variation (CV) of 6.4% at 2.04 ng/mL and 4.4% at 22.5ng/mL respectively, with a sensitivity of 0.5 ng/mL. Serum level of FSH, LH, progesterone and oestradiol were measured using commercially available Human ELSA kits for each parameter (32). The inter- and intra assay CV of 3.4% at 5.5 IU/L and 6.5% at 45.8 IU/L respectively, with a sensitivity of 1.5IU/L for FSH; inter- and intra-assay CV of 3.2% at 3.5 IU/L and 5.1% at 50.8 IU/L respectively, with a sensitivity of 0.25UI/L for LH; inter- and intra-assay CV of 4.8% at 10.5 nmol/L and 2.9% at 15.5 nmol/L respectively, with a sensitivity of 0.15nmol/L for progesterone, and inter- and intra-assay CV of 4.5% at 500.4pmol/L and 2.7% at 110.8 pmol/L respectively, with a sensitivity of 5.5pmol/L for estradiol.

### 3.3. Statistical Analysis

Statistical analysis was performed using SPSS version 11 software . All data were expressed as mean ± SD. One-way ANOVA; paired-sample and independent-sample t tests were applied where appropriate. Correlations were performed by Pearson's method. P < 0.05 was considered statistically significant in all comparisons.

## 4. Results

The BMI and waist/hip circumference ratio were calculated in addition to the measurement of blood pressure of subjects in both age groups in order to eliminated factors that may affect the level of serum leptin. BMI of subject was < 24, waist/hip circumference ratio was also < 0.8 and they were normotensive as presented in Table 1.

**Table 1:** Mean Value of Clinical Parameters that Measured and Their P Values Between Two Age Groups

	18-30 years	31-40 years	*P*
**Height, cm**	169.01 ± 2.4	170.12 ± 2.1	0.9317
**Weight, kg **	65.73 ± 1.3	66.85 ± 2.3	1.0219
**BMI**	23.01 ± 0.3	23.10 ± 0.6	1.2165
**Waist Cirumference, cm**	68.84 ± 1.9	71.03 ± 2.5	0.9213
**Hip Circumference, cm **	90.58 ± 2.7	91.92 ± 2.1	0.9410
**W/H ratio **	0.76 ± 0.01	0.77 ± 0.02	0.8967
**Systolic BP, mmHg **	115.74 ± 4.09	121.15±3.29	0.6323
**Diastolic BP, mmHg **	69.35 ± 2.17	70.43 ± 1.52	0.9017

Serum level of leptin, LH, FSH, progesterone and estradiol were measured on days 1, 7, 14, 21, and 28 of the menstrual cycle of subjects in both age groups. On day 14 and day 21 of the menstrual cycle, the mean leptin levels were significantly increased (P < 0.05) among age group 18-30 years compared to age group 31-40 years. On day 14 and day 28 of the cycle, the mean FSH levels were significantly reduced (P < 0.05) among age group 18-30 years compared to age group 31-40 years. On day 7 of the cycle, the serum level of LH was significantly increased (P < 0.05) among age group 18-30 years compared to age group 31-40 years. On day 21 of the cycle, serum progesterone level was significantly increased (P < 0.05) among age group 18-30 years compared to age group 31-40 years; however, on day 28 serum progesterone level was significantly reduced (P < 0.05) among age group 18-30 years compared to age group 31-40 years. On day 14 and day 21 of the cycle, the serum level of estradiol were significantly increased (P < 0.05) among age group 18-30 years compared to age group 31-40 years; leptin and fertility hormone results were presented on Table 2. Correlation analysis was performed on our data to see if there is any relationship between the levels of leptin in both age groups and the fertility hormones. Leptin was positively correlated with FSH on day 14; leptin was also positively correlated with LH on day 7 and day 21 for both age groups. On day 21 and day 28, leptin was positively correlated with progesterone, also with estradiol on day 7 and day 14 for both age groups; correlation analyses were presented on Table 3.

**Table 2:** Mean Serum Concentrations of Leptin, FSH, LH, Estradiol, Progesterone and Their P values Between Two Age Groups

	Days of Menstral Cycle	Age Groups		*P* value
		18-30 years	31-40 years	
**Leptin, ng/mL**				
	1	9.97 ± 1.6	9.35 ± 1.5	0.0624
	7	11.63 ± 5.4	11.21 ± 2.7	0.8946
	14	12.75 ± 5.8	11.60 ± 3.2	0.0371 a
	21	12.91 ± 3.2	11.98 ± 1.7	0.0413 a
	28	9.86 ± 1.2	9.09 ± 1.1	0.0518
**FSH, IU/L**				
	1	3.95 ± 0.9	4.03 ±1.2	0.1901
	7	5.81 ± 1.7	5.98 ± 1.5	0.1015
	14	7.02 ± 2.9	7.93 ± 2.6	0.0414 a
	21	5.31 ± 2.1	5.94 ± 1.9	0.0746
	28	4.01 ± 1.6	4.85 ± 2.8	0.0473 a
**LH**				
	1	5.36 ± 2.5	5.47 ± 2.1	0.1874
	7	8.60 ± 3.4	7.63 ± 1.9	0.0367 a
	14	24.32 ± 4.3	23.97 ± 5.1	0.0541
	21	9.71 ± 4.7	10.10 ± 5.3	0.0538
	28	5.01 ± 1.5	4.92 ± 0.9	0.2071
**Progesterone, nmol/L**				
	1	0.56 ± 0.1	0.66 ± 0.4	0.0713
	7	0.75 ± 0.8	0.83 ± 0.5	0.1830
	14	3.72 ± 1.2	4.01 ± 1.8	0.1096
	21	7.31 ± 1.4	5.38 ± 1.9	0.0382 a
	28	1.25 ± 1.1	1.98 ± 0.8	0.0410 a
**Oestradiol, pmol/L**				
	1	220.09 ± 38.9	221.59 ± 34.8	0.1093
	7	291.40 ± 45.5	275.12 ± 42.4	0.0439 a
	14	476.07 ± 73.1	432.97 ± 65.2	0.0331 a
	21	296.68 ± 40.7	298.88 ± 31.8	0.1174
	28	214.63 ± 31.7	204.69 ± 29.3	0.0764

^a^Significant level at (P < 0.05)

**Table 3:** Correlation of Serum Leptin Level With Individual Variable During the Normal Mmenstrual Cycle for Subjects Between Age Group (18-30 years) and (31-40 years)

	18-30 years		31-40 years	
	r	*P* value	r	*P* value
**FSH**				
**Day 1**	0.1472	0.1052	0.1039	0.2897
**Day 7**	-0.2154	0.0873	-0.1734	0.1108
**Day 14**	0.3124	0.0231 a	0.3157	0.0209 a
**Day 21**	-0.2098	0.0930	-0.2101	0.0851
**Day 28**	0.1964	0.1004	0.1016	0.2979
**LH**				
**Day 1**	-0.2521	0.0782	-0.2675	0.0621
**Day 7**	0.3627	0.0201 a	0.3485	0.0298 a
**Day 14**	0.1074	0.2809	0.1107	0.2601
**Day 21 **	0.3751	0.0179 a	0.3805	0.0159 a
**Day 28 **	-0.2580	0.0605	-0.2616	0.0598
**Progesterone**				
**Day 1**	-0.1893	0.1096	-0.1907	0.1041
**Day 7**	-0.1736	0.1102	-0.1861	0.1092
**Day 14**	0.2513	0.0791	0.2197	0.0895
**Day 21 **	0.4972	0.0025 b	0.4312	0.0075 b
**Day 28 **	0.3138	0.0210 a	0.3351	0.0192 a
**Oestradiol**				
**Day 1**	-0.1053	0.2874	-0.1109	0.2942
**Day 7**	0.3089	0.0267 a	0.3075	0.0289 a
**Day 14**	0.3456	0.0209 a	0.317	0.0212 a
**Day 21 **	0.1913	0.1065	0.1072	0.2015
**Day 28 **	-0.1744	0.1100	-0.1820	0.1099

^a^Significant level at the (P < 0.05)

^b^Significant level at the (P < 0.01)

## 5. Discussion

The present study was conducted to evaluate the relationship between circulating level of leptin at different phases of monthly menstrual cycles in fertile women. according to the results, we recorded a significant increased in serum leptin levels on day 14 (ovulatory phase) and day 21 (luteal phase) of the menstrual cycle in both age groups, this is an indication that estradiol and progesterone may play an important role in the regulation of leptin release during the normal menstrual cycle and this was in agreement with the study this finding was in agreement with the study conducted by Hardie et al.(33) they reported that circulating leptin levels rise during the transition from the follicular to the preovulatory phase and peak in the luteal phase at values more than 1.5-fold higher than those observed in the follicular phase which coincide with elevation of serum progesterone level. Similarly, Ludwig et al. (27) in their study reported that a considerable variation in leptin levels throughout the menstrual cycle, with higher levels in midluteal rather than the follicular phase. We recorded a low level of circulating leptin during the menstrual/secretory phase which corresponds to day 1/day 28 of the menstrual cycle for our subjects in both age groups. In contrast, Stock et al.(34) found a small overall variation in leptin levels during the menstrual cycle but when the phases were compared with each other; no statistically significant differences was obtained. The observed variation in leptin levels between phases seems to be influenced by a factor or factors on leptin expression. However, the significance increased of serum leptin level in the ovulatory phase and luteal phase in the present study may be connected to the fact that leptin may have a role in preparing the body for the metabolic demands of pregnancy (35).

The physiological rise of preovulatory eostradiol and midluteal progesterone appeared to have an influence on leptin production (33). In the present study, comparing the pattern of hormonal fluctuations throughout the menstrual cycle, leptin and progesterone show similar patterns of fluctuation, with increase in serum concentrations on days 14 and 21. This suggests a common stimulation factor for progesterone and leptin secretion. A previous study had suggested a stimulatory effect of progesterone on leptin secretion, which may be responsible for the fluctuations in leptin levels during the menstrual cycle (33). However, Mannucci et al. (22) showed no correlation in their study and suggested that progesterone has no effect on leptin secretion. The differences in finding can be attributed to the considerable differences in the number of patients included in their study, also differences in the assay methodologies; while on our own part we used the more recently validated and highly sensitive ELISA The literatures on the relationship between leptin and fertility hormones are inconsistent and the relationship appears to be complex.

In addition, a relationship between estrogen and leptin has been described during the follicular phase of both spontaneous menstrual cycles and menstrual cycles stimulated with exogenous FSH (33). This finding was in conformity with our present study, with increase in serum level of leptin on day 7 (proliferative/follicular phase) and a positive correlation between leptin and oestradiol; which suggests that leptin either has a direct effect or is regulated by gonadal steroids in the human ovary.

Recently, a study reported that release patterns of leptin and LH are synchronized. At night, as leptin levels peak, the pulsatory profile of LH becomes synchronous with that of leptin. It was proposed that leptin may regulate the minute-to-minute oscillations of the levels of LH and oestradiol, and that leptin may in this way determine the change in LH profile preceding ovulation (36).

In the present study because we recorded a positive correlation between serum level of LH and serum circulating leptin in both age groups during the preovulatory phase. Our findings in this study is in agreement with some of the studies as cited above but the conflicting results could be due to considerable individual variability of leptin production from the ob gene of adipocytes and various influences of other endocrine factors during the menstrual cycle. Ovarian steroid hormones might also affect plasma leptin concentrations through their action on adipose tissue. It has been demonstrated that oestradiol promotes the replication of adipose cells in culture (37). Fat tissues which contains abundant estrogen receptors represents15% of the body weight in a normal fertile woman (38). Therefore, the adipocytes constitute a dynamic endocrine/ metabolic compartment and are influenced by the biological expression of estrogen (39). In order to avoid the influence of adipose tissue and to be sure that variation in leptin levels was not affected by BMI; obese (BMI ≥ 24) subjects were excluded from this study, and therefore the effect of obesity on leptin fluctuation should have been eliminated.

When we compared the level of leptin in both age groups we discovered that there was significant increase in the level of serum leptin in women within age group (18-30 years) than those in age group (31-40 years). Since fertility tends to decrease from 31years and above, the higher level of leptin during ovulatory and luteal phase of the menstrual cycle in the younger age groups compared to older age groups is a pointer to interrelationship between fertility and leptin.

Our results suggest that there is a link between serum leptin and fertility hormone concentrations during the menstrual cycle, and the variation in circulating fertility hormones concentrations may have an influence in circulating leptin in female subjects of reproductive age. It is said that fertility is age related. From the age of 31 years and above, ease of fertility tends to decrease. The significant increase in circulating serum leptin level in the fertile younger age group and the correlation between leptin and fertility hormones left us with question whether leptin has any role to play in fertility?. Our finding in this study suggests that there may be a link between leptin levels and fertility. However, further studies are needed to prove if this association represents a cause-and-effect relationship.
